# Application of deep learning in evaluating the anatomical relationship between the mandibular third molar and inferior alveolar nerve: A scoping review

**DOI:** 10.4317/medoral.27584

**Published:** 2025-10-17

**Authors:** Suji Ahn, Min-Ji Kim, Jun-Young Kim, Wonse Park

**Affiliations:** 1 Department of Advanced General Dentistry, Yonsei University College of Dentistry, Seoul, Republic of Korea; 2 Department of Oral and Maxillofacial Surgery, Yonsei University College of Dentistry, Seoul, Republic of Korea; 3 Institute for Innovation in Digital Healthcare, Yonsei University, Seoul, Republic of Korea

## Abstract

**Background:**

With advancements in deep learning-based dental imaging analysis, artificial intelligence (AI) models are increasingly being employed to assist in mandibular third molar surgery. However, a comprehensive overview of the clinical utility remains limited. This scoping review aimed to identify and compare deep learning models used in the radiographic evaluation of mandibular third molar surgery, with a focus on AI model types, key performance metrics, imaging modalities, and clinical applicability.

**Material and Methods:**

Following the PRISMA-ScR guidelines, a comprehensive search was conducted in the PubMed and Scopus databases for original research articles published between 2015 and 2024. Systematic reviews, editorial articles, and studies with insufficient datasets were excluded. Studies utilising panoramic radiographs and cone-beam computed tomography (CBCT) images for AI-based mandibular third molar analyses were included. The extracted data were charted according to the AI model types, performance metrics (accuracy, sensitivity, and specificity), dataset size and distribution, validation processes, and clinical applicability. Comparative performance tables and heat maps were utilised for visualisation.

**Results:**

Of the initial 948 articles, 16 met the inclusion criteria. Various convolutional neural network (CNN)-based models have been developed, with U-Net demonstrating the highest accuracy and clinical utility. Most studies employed panoramic and CBCT images, with U-Net outperforming other models in predicting nerve injury and evaluating extraction difficulty. However, substantial variations in dataset size, validation procedures, and performance metrics were noted, highlighting inconsistencies in model generalisability.

**Conclusions:**

Deep learning shows promising potential in the radiographic evaluation of mandibular third molars. To date, most studies have relied on two-dimensional images and focused on detection and segmentation, while predictive modeling and three-dimensional CBCT-based analysis are relatively limited. To enhance clinical utility, larger standardized datasets, transparent multi-expert annotation, task-specific benchmarking, and robust external/multicenter validation are needed. These measures will enable reliable pre-extraction risk prediction and support clinical decision-making.

## Introduction

Third molar extraction is among the most frequently performed procedures in oral and maxillofacial surgery. Sensory disturbances involving the lower lip and chin, caused by the injury to the inferior alveolar nerve (IAN), are rare complications that may impair the patients' quality of life. Among several contributing factors, such as patient age, surgeon experience, traumatic tissue handling, and postoperative swelling, the proximity of the tooth root to the inferior alveolar nerve canal has been identified as the most significant (1 - 3).

Artificial intelligence (AI)- and deep learning-based diagnostic and predictive modelling methods have demonstrated utility in addressing complex clinical problems (4 - 8). Several recent studies have explored deep learning in clinical dentistry, particularly in analysing panoramic radiographs and cone-beam computed tomography (CBCT) images to predict the risk of inferior alveolar nerve damage or evaluate extraction difficulty based on the positional relationship between the third molar and the mandibular canal (9 - 24).

However, despite advances in deep-learning-based dental imaging analysis, comprehensive evaluations of the clinical benefits of these models remains limited. This scoping review aimed to systematically map the AI models employed for mandibular third molar image analysis and to evaluate their potential clinical advantages.

## Material and Methods

A comprehensive literature search was conducted using the PubMed and Scopus databases to identify relevant studies published between 1 January 2015 and 31 December 2024. The search strategy was formulated to capture studies employing deep learning models for assessing the mandibular third molar and IAN using radiographic imaging. The following search query was employed: ("deep learning" [Title/Abstract] OR "inferior alveolar nerve" [Title/Abstract] OR "artificial intelligence" [Title/Abstract] OR "radiography" [Title/Abstract]) AND ("dental" [All Fields] OR "third molar" [All Fields]) AND ("image" [Title/Abstract] OR "analysis" [All Fields]) AND ("detection" [All Fields] OR "classification" [All Fields] OR "segmentation" [All Fields] OR "prediction" [All Fields]). No restrictions were applied to the study design, provided the studies met the predefined eligibility criteria.

The studies were selected based on predefined inclusion and exclusion criteria to ensure relevance and methodological rigor. Two independent reviewers initially screened the articles by evaluating their titles and abstracts. Full-text articles were subsequently assessed for final inclusion according to the eligibility criteria. Disagreements between reviewers were resolved through discussion; and if necessary, a third reviewer was consulted.

Studies were included if they used deep learning models for mandibular third molar and IAN assessment, employed panoramic radiographs or CBCT as imaging modalities, reported quantitative performance metrics such as accuracy, sensitivity, specificity, and area under the curve (AUC); and were published in English within the defined timeframe. Studies were excluded if they applied non-deep learning methodologies, did not directly pertain to mandibular third molar assessment, or were categorised as review articles, case reports, editorials, or conference abstracts.

**Figure 1:**
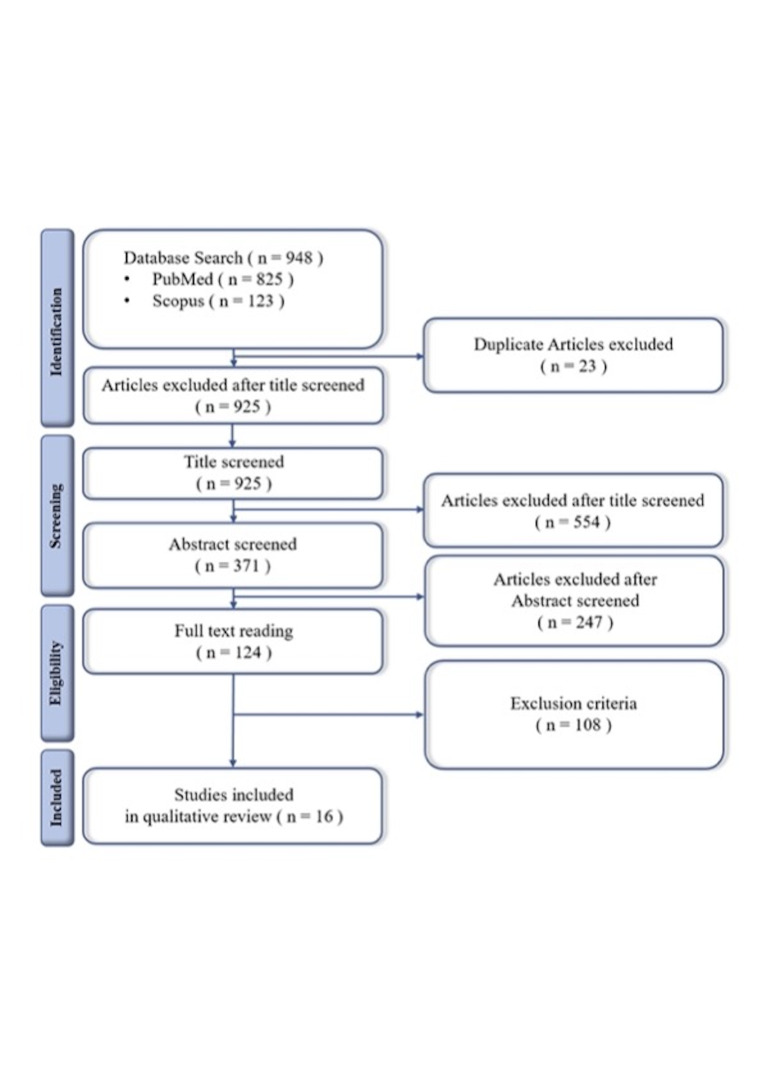
Study selection flow chart.

The study selection process is illustrated in the PRISMA 2020 flow diagram (Figure 1). A total of 948 articles were identified from PubMed (n=825) and Scopus (n=123). After the removing 23 duplicate records, 925 unique records were retained for screening. After title and abstract screening, 554 records were excluded for not meeting the eligibility criteria. Subsequently, 371 reports were retrieved in full-text and all were successfully obtained. After full-text assessment, 124 reports were reviewed for eligibility, of which 108 were excluded due to insufficient data (n=68), irrelevance to the study topic (n=25), lack of radiographic imaging (n=10), or unavailability of the full text (n=1). Sixteen studies met the inclusion criteria, and were included in the final systematic review.

1

Data charting was conducted using a standardised data extraction form calibrated beforehand to ensure consistency and accuracy. The extraction form included predefined categories, such as study characteristics (author, publication year, and country), methodological details (study design, dataset size, and imaging modality), deep learning model information (architecture and training parameters), performance metrics (accuracy, sensitivity, specificity, and AUC), and clinical applicability.

Two independent reviewers charted the data in duplicate to minimise errors and biases. Any discrepancies were resolved through discussion, and a third reviewer was consulted, if necessary. This approach ensured high reliability of data extraction and reduced subjectivity in data interpretation. In cases of unclear or missing information, the corresponding authors of the included studies were contacted for further clarification.

## Results

**Figure 2:**
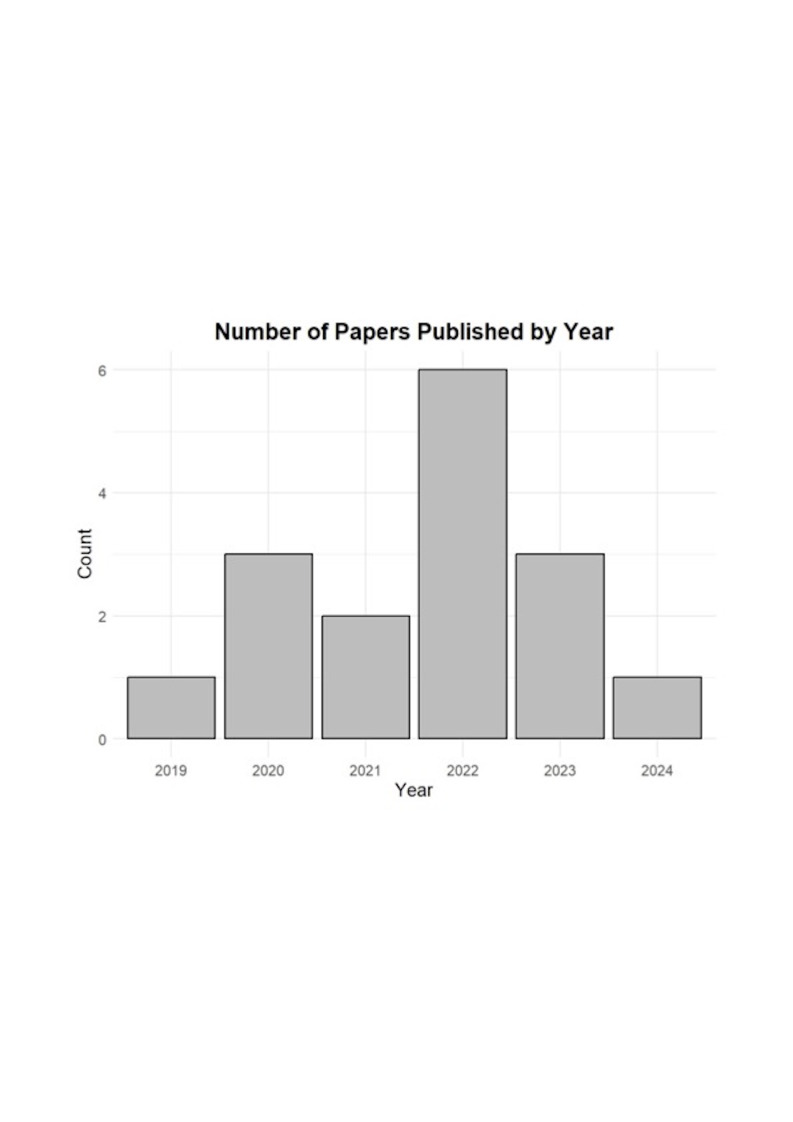
Number of papers published annually.

Among the 16 articles reviewed, a notable increase was observed by 2022 in studies evaluating deep learning models for detecting and classifying the impaction status of the third molar, assessing its positional relationship with the mandibular canal, and predicting the risk of IAN injury (Figure 2).

2

Deep learning models play a crucial role in evaluating the positional relationship between the mandibular third molar and the IAN, as well as in assessing the risk of nerve injury. To systematically analyse, this study categorised AI models according to their imaging analysis approaches and objectives.

**Figure 3:**
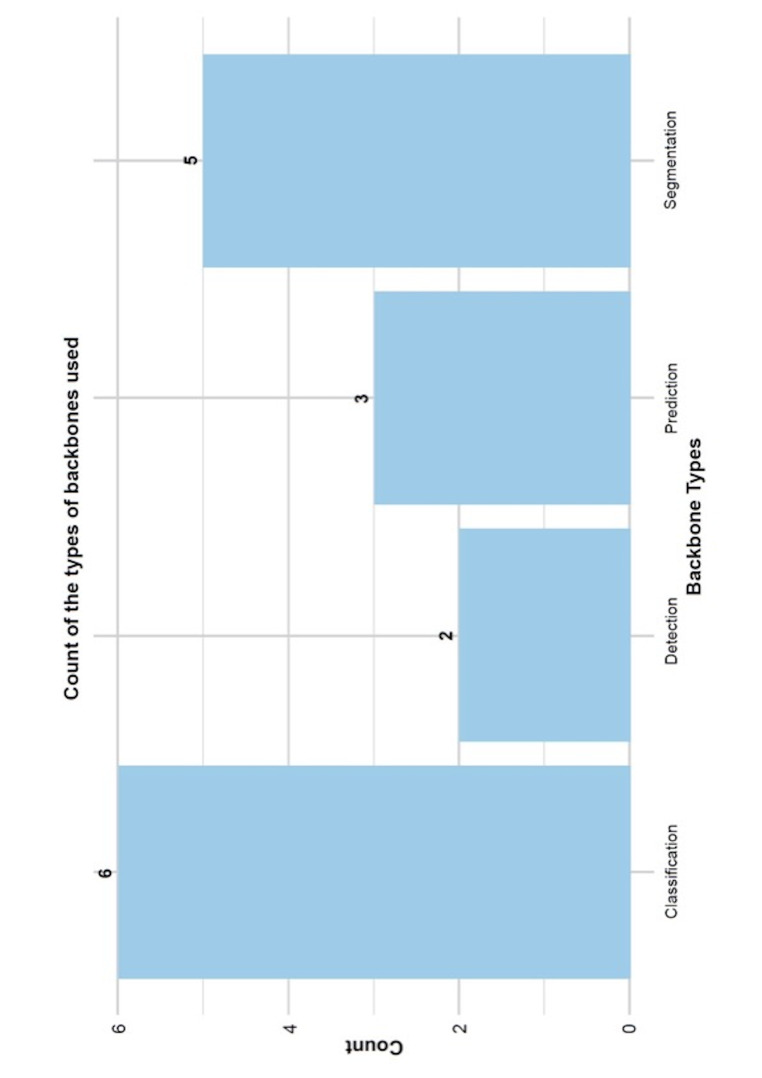
Types of backbones used for data analysis in the included papers.

The AI models were applied to four principal domains of image analysis. Some models focused on determining the presence and impaction status of the third molar, whereas others were designed to identify its spatial relationship with the IAN. Additionally, segmentation techniques were employed to delineate anatomical structures at the pixel level, thereby offering more detailed spatial information. Predictive models were used to assess extraction difficulty and estimate the risk of nerve injury, thereby supporting clinical decision-making. The distribution of model backbones across the included studies is summarized in Figure 3.

3

Table 1-4 summarise the characteristics and training methodologies of various AI models, thereby facilitating a comparative analysis of their applications in assessing mandibular third molar. These tables offer a comprehensive overview of the utilisation of different AI techniques in radiographic image analysis, aiding in the identification of key patterns across different studies.

Table 1 provides an overview of the classification models employed to detect the presence and impaction status of mandibular third molars. The most frequently utilised architectures comprised MobileNet-V2, VGG-16, and ResNet-50 with dataset sizes ranging from 500 to 1,330 images. Multiple data augmentation techniques, such as image rotation, flipping, and resizing, were applied to enhance model generalisability. Classification models were frequently integrated with detection and segmentation models to strengthen multitask learning frameworks.

Table 1

Table 2 summarises object detection models used to automatically localise mandibular third molars and analyse their spatial relationships with the IAN. Commonly employed architectures include YOLOv3, ResNet-50, and VGG-16, with dataset sizes ranging from 440 to 579 panoramic images. Data augmentation techniques, such as image rotation and flipping, were frequently applied to improve model robustness. Detection models are essential for preoperative risk assessment, as they accurately delineate third molar boundaries and estimate proximity to the IAN.

Table 2

Table 3 outlines segmentation models employed to delineate anatomical structures such as the mandibular canal and third molars, at the pixel level. The U-Net architecture was most frequently used, featuring in five studies due to its up-sampling and skip connection mechanisms, which facilitate precise segmentation despite limited datasets. Other architectures, such as SegNet and 3D U-Net, were applied in selected cases. Dataset sizes varied considerably, ranging from 81 to 3,200 images, with data augmentation techniques, such as image rotation, scaling, and elastic deformation, enhancing model generalisation. Segmentation models demonstrated high accuracy in outlining the mandibular canal and third molars, with Dice coefficients ranging from 0.80 to 0.94. These findings suggest that AI-based segmentation may significantly enhance diagnostic precision during third molar extractions.

Table 3

Table 4 summarises predictive models developed to assess extraction complexity and risk of IAN injury. Frequently utilised architectures included ResNet-34, EfficientDet-D4, YOLOv3, and YOLOv8, with dataset sizes ranging from 600 to 2,394 panoramic radiographs. To optimise prediction accuracy, various data augmentation strategies, including brightness and contrast adjustments, image scaling, and region-of-interest modifications, were employed.

Table 4

**Figure 4:**
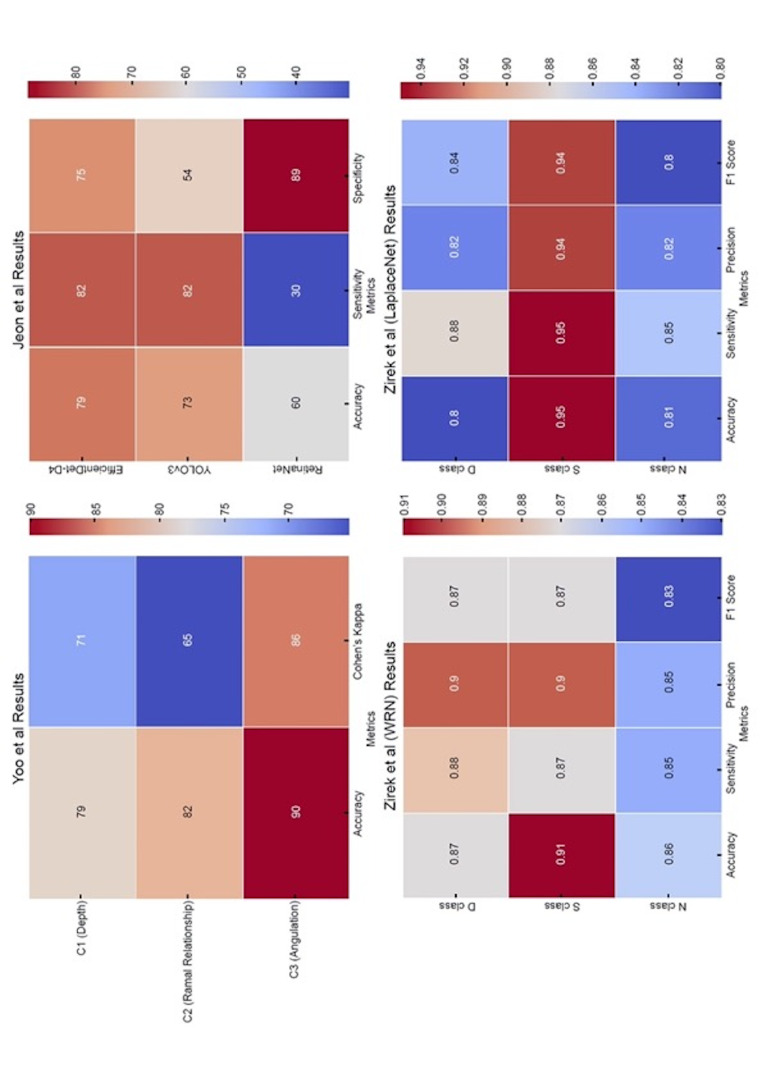
Heatmaps of deep learning model performance regarding prediction.

Key predictive performance metrics-including accuracy, sensitivity, specificity, precision and F1 score-are consolidated in Figure 4, which serves as a critical reference for assessing clinical applicability.

Studies employing predictive models demonstrated relatively high performance, with certain models achieving &gt;80% accuracy in predicting extraction difficulty and the risk of IAN injury. Such models offer clinically meaningful insights that support preoperative decision-making and risk stratification.

Deep-learning models have exhibited high reliability in predicting extraction difficulty and the potential for mandibular nerve injury in multiple studies. For example, in a study conducted by Yoo et al. (22), the models predicted the depth, ramal relationship, and angulation of the mandibular third molar with accuracies of 78.91%, 82.03%, and 90.23%, respectively, and Cohen's kappa values ranging from 65.23 to 85.54. These findings suggest that a predictive model integrating depth, angulation, and ramal relationship can serve as a reliable tool in clinical settings (Figure 4). Jeon et al. (23) evaluated the prediction of extraction difficulty and mandibular nerve damage by comparing the EfficientDet-D4, YOLOv3, and RetinaNet models and reported that the EfficientDet-D4 model exhibited the highest performance (accuracy, 78.65%; sensitivity, 82.02%) (Figure 4), indicating its clinical potential for precise preoperative evaluation.

4

To ensure comparability across studies, specific assumptions were applied when necessary. Studies reporting multiple performance metrics for various models or configurations were standardised by selecting the most clinically pertinent metrics (accuracy for overall classification and AUC for risk prediction tasks). In cases where the sensitivity and specificity values were not explicitly reported, these values were calculated based on the available confusion matrix data, wherever possible.

An essential task in developing a predictive model is to minimise both false-positive and false-negative results by maintaining a balance between sensitivity and specificity. Zirek et al. (24) reported promising performance metrics for models such as WideResNet and LaplaceNet, with accuracies ranging from 80-91%, depending on the specific task (Figure 4). However, the reported precision and recall values varied, suggesting that further optimisation is necessary for the clinical application of these models.

A structured critical appraisal was conducted using a modified Joanna Briggs Institute (JBI) checklist for diagnostic and prognostic studies. This evaluation focused on study design, methodological rigour, dataset quality, model performance evaluation, and risk of bias. Each study was scored according to these criteria and those with significant methodological limitations were interpreted cautiously during data synthesis. The appraisal findings were discussed to highlight the strengths and limitations of the existing literature.

Excluding five studies, the remainder employed data augmentation techniques, such as image flipping, rotation, and scaling, to enhance model generalisability and reduce overfitting. Experts conducted annotations for labelling standards, region-of-interest identification, and segmentation tasks, applying various deep-learning algorithms depending on the specific task. More than 50% of these studies provided detailed information regarding the annotators (clinical experts and radiologists) and the annotation protocols employed. Several studies demonstrated annotation robustness by involving multiple annotators and incorporating consensus or correction steps to ensure accuracy. Despite high performance on controlled datasets, real-world clinical application remains challenging due to anatomical variability and inconsistencies in image quality.

The extracted data were synthesised using descriptive and visual analyses. Descriptive statistics, including means and standard deviations, were utilised to summarise performance metrics across various AI models. The key findings for predictive tasks are presented as a comparative heat map to facilitate pattern recognition and trends in performance (Figure 4).

Furthermore, the reported limitations of the included studies, particularly dataset heterogeneity and external validation constraints, were analysed to inform recommendations for future research. These findings were incorporated into the

## Figures and Tables

**Table 1:** Table Characteristics of AI models used in the included studies according to the object detection method: Classification.

Author		Neural network architecture	Backbone	Data set used to develop the AI model (Number of datasets)	Testing data set	Training and validation datasets	Data augmentation
Classification							
2021	Vinayahalingam et al.	CNN	MobileNet -V2	500 cropped panoramic radiographs(PR) were used to classify carious lesions in mandibular and maxillarythird molars	100 images	400 images	Image rotationFlippingResizing
2022	Sukegawa al.	CNN	VGG-16	1,330 images of mandibular third molars obtained from digitalradiographs taken at the Department ofOral and Maxillofacial Surgery at a general hospital between 2014 to 2021.	Not specified	Not specified	Not specified
2022	Choi et al.	CNN	ResNet-50	571 panoramic images of mandibular third molars	428 images	143 images	Image rotationHorizontal flippingBrightness adjustment
2022	Sukegawa et al.	CNN	ResNet-50 and ResNet50V2	1,279 images of mandibular third molars obtained from patients who visited the Department of Oral andMaxillofacial Surgery at a general hospital between 2014 and 2021	128 images (10% of 1279 images)	1151 images(90% of 1279 images)	Random rotation within the 18 to 18 degrees rangesRandom horizontal and vertical flippingRandom translation within a range of 30 pixels
2023	Kempers et al.	CNN	MobileNet-V2	863 preoperative panoramic radiographs of patients who underwentthird molar extraction surgery between2019 and 2020	130 panoramic radiographs including 217Images of mandibular third molars	773 panoramic radiographs	Not specified
2023	Youn Kim et al.	CNN	WideResNet and LaPlaceNet	1000 panoramic images	Winter's classification:200 radiographsAll impacted teeth detection:240 radiographs	Winter's classification: 1800 radiographsAll Impacted TeethDetection: 2154 radiographs	Random rotationHorizontal and vertical flippingRandom scaling Translation

1

**Table 2:** Table Characteristics of the AI models used in the included studies according to the object detection method: Detection.

Author		Neural network architecture	Backbone	Data set used to develop the AI model (Number of datasets)	Testing data set	Training and validation datasets	Data augmentation	

				Detection				
2022	Takebe et al.	CNN	YOLOv3	579 panoramic images	96 images	483 images	Image rotation Flipping	

2022	Celik	CNN	RCNN, ResNet-50, VGG16 and YOLOv3	440 panoramic images	Not specified	Not specified	Rotation by 5 degrees Horizontal flipping	


2

**Table 3:** Table Characteristics of the AI models used in the included studies according to the object detection method: Segmentation.

Author		Neural network architecture	Backbone	Dataset used to develop the AI model	Testing dataset	Training and validation datasets	Data augmentation	

				Segmentation				
2019	Vinayahalingam et al.	CNN	U-net	81 dental panoramic images	30 images	70 images	Image rotationScaling and croppingColor transformation	


2020	Jaskari et al.	CNN	U-net	637 cone beam CT volumes	128 cone beam CT volumes	509 cone beam CT volumes	Random rotationHorizontal and vertical flippingRandom scalingElastic deformation	



2020	Orhan et al.	CNN	U-net	130 cone beam CT volumes	Not specified	Not specified	Not specified	
2020	Kwak et al.	CNN	U-net	100 cone beam CT volumes	20 cone beam CT volumes	80 cone beam CT volumes	Not specified	
2022	Ariji et al.	CNN	U-net	3200 dental panoramic images	1380 images	881 images	Not specified	

3

**Table 4:** Table 4

Author		Neural network architecture	Backbone	Dataset used to develop the AI model	Testing data set	Training and validation datasets	Data augmentation	

				Prediction				
2021	Yoo et al.	CNN	ResNet-34	600 preoperative panoramic radiographsincluding 1053 images ofthird molars	Not specified	Not specified	Perform image flipping with a probability of 0.5Randomly adjust the image scale within the range of (0.8, 1.0)Randomly select brightness and contrast adjustment factors within the range of (0.8, 1.2)Edit ROI within the scale range of (0.9, 1.0)	



2023	Jeon et al.	CNN	RetinaNetYOLOv3EfficientDet-D4	901 panoramic images	178 images	723 images	Image RotationHorizontal FlipRandom scalingAdjust Brightness	



2024	Zirek et al.	CNN	YOLOv8	For Winter's classification: 2000 radiographsFor All Impacted TeethDetection: 2394 radiographs	For each dataset, 10% of the total data	For each dataset,90% of the total data	Image RotationHorizontal FlipRandom scaling Translation	




4

## Data Availability

The datasets used and/or analyzed during the current study are available from the corresponding author.
